# Single breathhold 3D balanced SSFP coronary MRA at 3.0T: a reproducibility study

**DOI:** 10.1186/1532-429X-15-S1-E11

**Published:** 2013-01-30

**Authors:** Sahar Soleimanifard, Matthias Stuber, Allison Hays, Robert G Weiss, Michael Schär

**Affiliations:** 1 Johns Hopkins University, Baltimore, MD, USA; 2 CIBM & University of Lausanne, Lausanne, Switzerland; 3 Philips Healthcare, Cleveland, OH, USA

## Background

Balanced steady-state free precession (bSSFP) imaging [1] has developed as the method of choice for coronary MRA at 1.5T. The bSSFP acquisition, however, is especially susceptible to static magnetic field (B0) inhomogeneities and radio frequency transmit field (B1+) distortions, both of which are more pronounced at higher field strengths, resulting in degraded and variable image quality at 3.0T compared to 1.5T. Although modified bSSFP sequences have been recently developed [2], these modified implementations too come with notable drawbacks such as longer scan time and lower SNR compared with conventional bSSFP. Therefore, the adoption of bSSFP at 3.0T is still quite limited and it has led to reutilization of spoiled gradient echo techniques at 3.0T, sometimes at the expense of using contrast agents [3]. Recent advances in hardware and software, such as 32-channel receive coils, multi-transmit systems, and localized shimming, may however improve the performance of bSSFP at 3.0T. The purpose of this work was to develop an accelerated single breathhold bSSFP sequence at 3.0T taking advantage of these advances, and to test reproducibility of the implemented sequence.

## Methods

A three-dimensional, volume-targeted bSSFP sequence was implemented on a 3.0T MR scanner equipped with multi-transmit system and a 32-channel cardiac phased-array coil. Image-based shimming was performed using B0-map [4] and B1+-map [5,6] images to minimize field inhomogeneities. Parallel imaging (2.5 SENSE factor with 1.3 oversampling factor) was used to accelerate the acquisition. A half-alpha TR-half preparation pulse with 10 startup RF pulses was used to accelerate the transition to steady-state. Fat suppression was achieved using a spectrally selective saturation pulse. Scan parameters were the following: TR/TE 3.9/1.9 ms, RF excitation angle 50°, field-of-view 300×300×20 mm^3^, reconstructed voxel size 0.8×0.8×1.0mm^3,^, acquisition window 105ms, scan time 20.5±2.0s. 15 healthy volunteers and 3 patients with coronary artery disease (CAD) were included (age 38±18 (21-75) years; 8 female), and the acquisition was repeated in 9 subjects. The vessel length, diameter, and sharpness were analyzed using the Soap-Bubble reformatting tool [7]. The intra-observer, inter-observer, and inter-scan reproducibilities were quantified using regression analysis and intra-class correlation coefficient, in a blinded manner.

## Results

**Figure 1:**
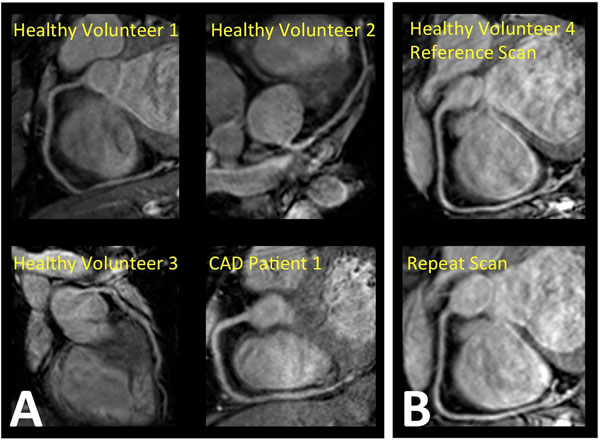
Single breathhold multi-planar reformatted bSSFP images of RCA and LAD in three healthy volunteers and one CAD patient (A). RCA images acquired in two examinations, in a healthy volunteer, demonstrating excellent visual reproducibility (B).

The bSSFP sequence provided uniform, high quality depiction of coronary arteries as shown in Figure 1. The average visible vessel length of 100.5±6.3mm and sharpness of 55±2% compared favorably with earlier reported navigator-gated bSSFP and gradient echo sequences at 3.0T [8]. As shown in Table 1, an excellent degree of reproducibility was obtained.

**Table 1:** Length, diameter, and sharpness of coronary arteries in 18 subjects (RCA = 15, LAD = 5)

	Intra-observer (n=20)			Inter-observer (n=20)		Inter-scan (n=9)		
	Reference analysis	Repeat analysis	ICC	Analysis	ICC	Reference analysis (subset)	Repeat scan analysis	ICC

Length [mm]	100.5±6.3	101.6±6.1	0.993	98.7±6.6	0.896	95.7±10.0	98.2±9.5	0.974

Diameter [mm]	2.8±0.1	2.8±0.1	0.987	2.8±0.1	0.976	2.7±0.1	2.8±0.1	0.961

Vessel Sharpness [%]	55±2	54±2	0.989	56±1	0.938	54±3	54±3	0.905

Data are presented as mean ± SEM. ICC = intra-class correlation coefficient. Diameter and vessel sharpness values are averaged in the entire visualized length of the coronary arteries.

## Conclusions

The refined 3D bSSFP acquisition, using a state-of-the-art MR scanner, allows accelerated, reproducible imaging of major coronary arteries during a single breathhold in healthy adult subjects and patients with CAD.

## Funding

NIH/NHLBI (ROIHL084186, ARRA 3R01Hl084186-04S1) and American Heart Association (12PRE11510006).
